# Sex differences in systematic screening for tuberculosis among antiretroviral therapy naïve people with HIV in Kampala, Uganda

**DOI:** 10.1186/s12879-025-10835-4

**Published:** 2025-04-01

**Authors:** Lelia H. Chaisson, Fred C. Semitala, Sandra Mwebe, Eileen P. Scully, Jane Katende, Lucy Asege, Martha Nakaye, Alfred O. Andama, Adithya Cattamanchi, Christina Yoon

**Affiliations:** 1 UCSF Center for Tuberculosis, University of California, San Francisco, San Francisco, CA USA; 2 UCSF Institute for Global Health Sciences, University of California, San Francisco, San Francisco, CA USA; 3 Department of Internal Medicine, Makerere University, Kampala, Uganda; 4 Infectious Diseases Research Collaboration, Kampala, Uganda; 5 Makerere University Joint AIDS Program, Kampala, Uganda; 6 Division of Infectious Diseases, Department of Medicine, Johns Hopkins University School of Medicine, Baltimore, MD USA; 7 Division of Pulmonary Diseases and Critical Care Medicine, Department of Medicine, University of California Irvine, Irvine, CA USA; 8 Division of Pulmonary and Critical Care Medicine, Department of Medicine, Zuckerberg San Francisco General Hospital and Trauma Center, University of California San Francisco, 1001 Potrero Ave., Room 5K1, 94110 San Francisco, USA

**Keywords:** Tuberculosis, HIV, Sex differences, Systematic screening, Symptoms, C-reactive protein

## Abstract

**Background:**

Systematic tuberculosis (TB) screening is recommended for all people with HIV (PWH) because of its potential to improve TB outcomes through earlier diagnosis and treatment initiation. As such, systematic screening may be particularly important for men, who experience excess TB prevalence and mortality compared to women. We assessed sex differences among PWH undergoing systematic TB screening, including TB prevalence and severity, diagnostic accuracy of screening tools, and TB outcomes.

**Methods:**

We enrolled and followed adults with HIV (CD4 ≤ 350 cells/µL) initiating antiretroviral therapy (ART) at two HIV/AIDS clinics in Uganda from July 2013 to December 2016. All participants underwent TB screening and sputum collection for TB testing (Xpert MTB/RIF [Xpert], culture). We evaluated diagnostic accuracy of four WHO-recommended TB screening strategies (symptom screen; C-reactive protiein [CRP]; symptom screen followed by CRP, if symptomatic [symptoms + CRP]; Xpert) for culture-positive TB and compared TB prevalence, days-to-treatment initiation, and 3-month mortality by sex.

**Results:**

Of 1,549 participants, 727 (46.9%) were male and 236 (15.2%) had culture-positive TB. Compared to females, males had lower pre-ART CD4 counts (median 139 vs. 183 cells/µL, *p* < 0.001), higher TB prevalence (20.5% vs. 10.6%, *p* < 0.001), and higher mycobacterial load as measured by Xpert semi-quantitative grade (*p* = 0.03). Sensitivity was high (≥ 89.8%) for all screening strategies except Xpert (Xpert sensitivity 57.2%) and did not differ by sex. Specificity varied widely from 13.9% for symptom screen to 99.2% for Xpert, and was 5–15% lower for males than females for symptom screen, CRP, and symptoms + CRP. Among PWH with culture-positive TB, median days-to-treatment initiation (2 vs. 4, *p* = 0.13) and 3-month mortality (9.4% vs. 9.2%, *p* = 0.96) were similar for males and females.

**Conclusions:**

Although ART-naïve males undergoing systematic screening had more advanced HIV and TB than females, days-to-TB treatment initiation and early TB mortality were similar, suggesting that systematic TB screening has the potential to reduce sex-based disparities in TB outcomes.

**Supplementary Information:**

The online version contains supplementary material available at 10.1186/s12879-025-10835-4.

## Background

In almost all settings, men with and without HIV shoulder a disproportionately higher tuberculosis (TB) burden than women. Globally, in 2021, the male-to-female ratio of notified TB cases was 1.7:1.0 [1]. This excess in TB among men has been confirmed by prevalence surveys, which use systematic screening to identify individuals with TB disease, thereby reducing care-seeking biases that can affect TB case notification data [2–4]. Data from TB prevalence surveys have also revealed higher prevalence-to-notification ratios among men in most settings, suggesting that men with TB are more likely to go undiagnosed and untreated than women. Delays in TB diagnosis and treatment initiation likely contribute to poorer TB outcomes experienced by men with TB/HIV, who have a 30% higher odds of death than women, and may result in ongoing TB transmission within families and communities [5]. Improving TB diagnosis and reducing time to treatment initiation for people with HIV (PWH) remains critical to reducing the global TB burden, and strategies to increase case detection among men are particularly needed.

World Health Organization (WHO) guidelines state that PWH should be systematically screened for TB at every clinical encounter using one of several recommended TB screening tools (WHO 4-part symptom screen, C-reactive protein [CRP], chest x-ray [CXR], WHO-recommended rapid molecular tests) [6]. Provider-initiated systematic TB screening is a key component of the WHO End TB Strategy [7, 8] and can lead to earlier TB diagnosis, improved TB outcomes, and reduced TB transmission compared to passive case finding, which relies on self-reported TB symptoms. However, biological, social, and behavioral differences between males and females may influence the yield and impact of systematic screening for TB among PWH. The diagnostic yield of systematic TB screening may be higher among men with HIV, who have been shown to delay entry to HIV care [3] and have more severe TB disease at the time of diagnosis compared to women with HIV [9]. Furthermore, differences in the prevalence and spectrum of both TB and non-TB disease have the potential to affect the performance of recommended TB screening tools. Notably, however, systematic screening has the potential to improve TB outcomes, particularly among men with HIV, by enabling earlier TB diagnosis and earlier treatment initiation, thereby reducing sex-based differences in TB morbidity and mortality.

Importantly, few systematic screening studies report sex-disaggregated data [10–14]. As such, the extent to which the yield and potential impact of systematic TB screening among PWH differs by sex, if any, remains unclear. Therefore, in this study, we aimed to assess sex differences in TB burden (prevalence, mycobacterial load), the diagnostic accuracy of four WHO-recommended TB screening strategies (symptom screen; CRP; symptom screen followed by CRP, if symptomatic; and Xpert MTB/RIF [Xpert]), and the impact of systematic TB screening (yield, time-to-treatment initiation and early mortality) among antiretroviral therapy (ART)-naïve adults with HIV in a high TB/HIV burden setting.

## Methods

### Study population

We performed a secondary analysis of participants enrolled in a prospective cohort study evaluating TB screening and confirmatory testing algorithms among consecutive outpatient PWH in Kampala, Uganda, conducted from July 2013 to December 2016 [15–19]. ART-naïve adults (≥ 18 years) with a pre-ART CD4 + T-cell count ≤ 350 cells/uL within three months of study enrollment were enrolled from two HIV clinics. Patients taking medication with anti-mycobacterial activity within three days of enrollment were excluded.

### Study procedures

*Baseline activities.* Trained study staff collected demographic and clinical data at enrollment. All participants underwent symptom screening using a standardized questionnaire, finger prick for CRP testing, and sputum collection for TB testing, regardless of TB screening test results. We considered participants to screen positive by symptoms if they reported at least one of four TB symptoms (current cough, fever, night sweats, weight loss) in the past 30 days. Baseline CRP levels were measured from capillary blood using a standard sensitivity point-of-care (POC) assay (iCHROMA CRP Reader, BodiTech Med Inc., South Korea). In accordance with WHO recommendations, we considered participants to screen positive by CRP if CRP was ≥ 5 mg/L [6]. We collected two spot sputum samples from all study participants for Xpert testing and liquid mycobacterial culture (BACTEC 960 Mycobacterial Growth Indicator Tube). Xpert testing was performed using ≥ 1 mL of sputum from the first specimen and mycobacterial culture was performed on decontaminated sediments from both sputum specimens [16]. Staff performing Xpert testing and culture were blinded to demographics, symptom screen status, and CRP concentrations.

*Follow-up activities.* Participants were followed for three months after enrollment. We assessed vital status for all patients in person or by telephone and determined dates of death by reviewing medical records and interviewing patients’ household members. In addition, we extracted dates of TB treatment initiation from chart review of clinic records.

### Reference standard

We considered participants to have prevalent TB if *M. tuberculosis* was isolated from one or more cultures. We considered patients to not have prevalent TB if all sputum cultures were negative for *M. tuberculosis*, with a minimum of two negative cultures.

### Statistical analysis

We compared baseline demographic and clinical characteristics, TB prevalence, measures of mycobacterial load at TB diagnosis (number and duration of TB symptoms, the proportion with Xpert-positive and culture-positive TB, Xpert semi-quantitative grade, and days-to-culture positivity), days-to-TB treatment initiation (if culture positive) and early mortality (defined as death within three months of enrollment), between males and females using Wilcoxon rank sum tests for continuous variables and Chi-square or Fisher’s exact tests for categorical variables. We calculated the point estimates and 95% confidence intervals for the sensitivity, specificity, and predictive values of the following four TB screening strategies for males and females, in reference to culture results: (1) symptom screening, (2) CRP, (3) symptom screening followed by CRP testing, if symptom screen-positive (screen positive defined as both tests positive) and (4) Xpert (Xpert-for-all). We compared differences in sensitivity and specificity between males and females using a two sample test of proportions. We used multivariable logistic regression to compare the odds of culture-positive TB between males and females, adjusting for a priori confounders and socio-behavioral and clinical factors associated with TB in unadjusted analyses.

### Ethics approval

This study was approved by the Institutional Review Boards of the University of California San Francisco, Makerere University School of Medicine Research Ethics Committee, and the Uganda National Council for Science and Technology. All participants provided written informed consent.

## Results

### Study population

**Fig. 1:**
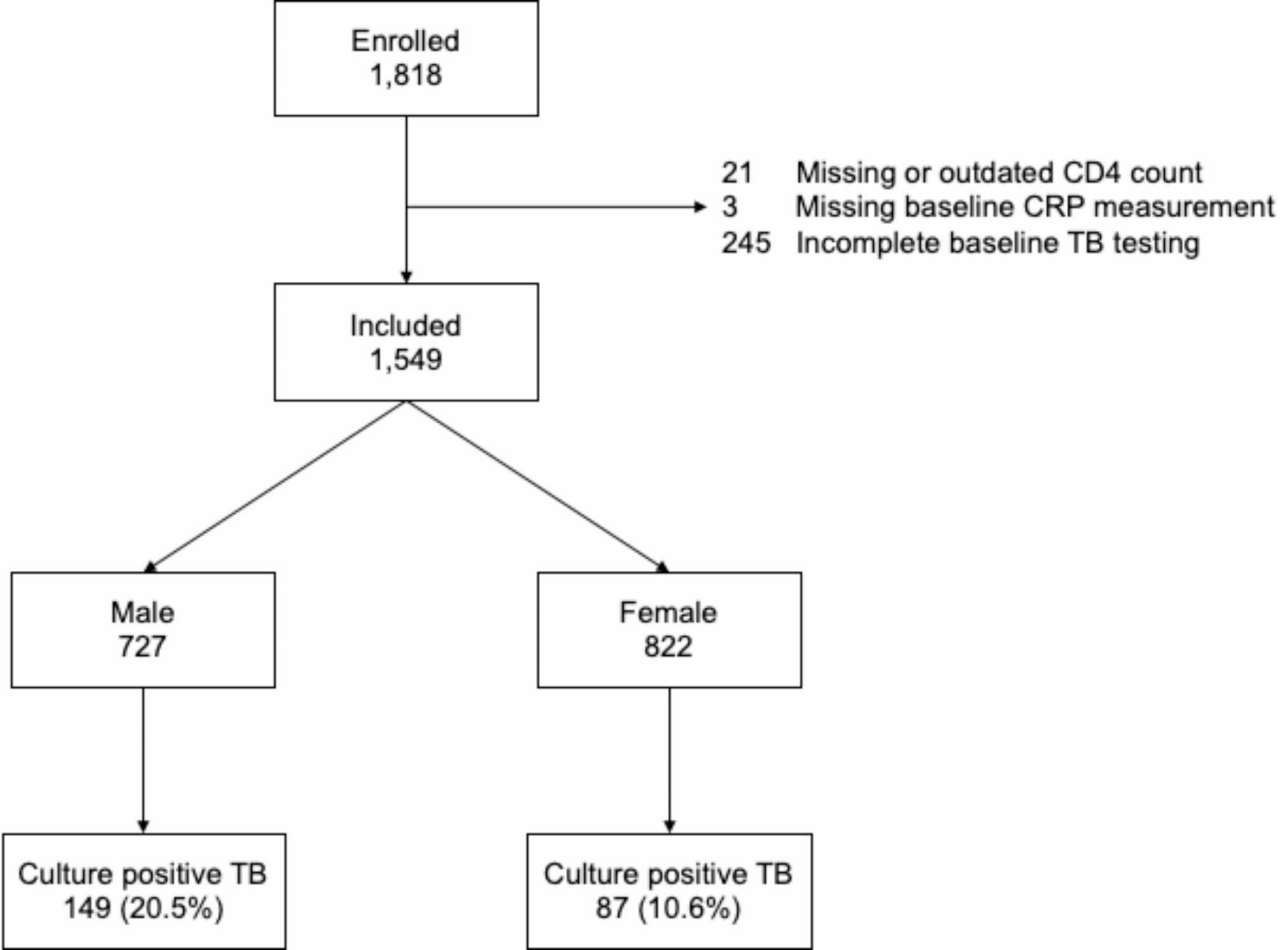
Participant flow diagram

From July 2013 through December 2016, we consecutively enrolled 1,818 eligible participants. Of these, we excluded 21 with missing or outdated CD4 counts, 3 with a missing baseline CRP measurement, and 245 with incomplete baseline TB testing (Fig. 1).

Of the 1,549 participants included in this analysis, 727 (46.9%) were male, median age was 33 years (IQR 27–40), median pre-ART CD4 count was 158 cells/µl (IQR 67–263), median BMI was 21.1 kg/m^2^ (IQR 18.9–23.9), and 55 (3.6%) had a history of prior TB (Table 1). Of the 822 females, 23 (2.8%) were pregnant at study enrollment; Supplementary Table 1 describes baseline characteristics of pregnant participants. Compared to females, males were older (median age 36 vs. 30 years, *p* < 0.001), had lower BMI (median 20.2 vs. 22.3 kg/m^2^, *p* < 0.001), lower CD4 counts (median 139 vs. 183 cells/µl, *p* < 0.001), and were more likely to report a history of prior opportunistic infection (46.2% vs. 39.1%, *p* = 0.01). Overall, 236 (15.2%) participants had culture-positive TB, including 149/727 (20.5%) males and 87/822 (10.6%) females (*p* < 0.001, Table 2). Adjusted for age, pre-ART CD4 cell count, BMI, temperature, heart rate, and smoking, males had a two-fold higher odds of culture-positive TB compared to females (adjusted odds ratio 2.05, 95% CI 1.40–3.01, *p* < 0.001, Table 3).

**Table 1:** Characteristics of ART-naive males and females with HIV undergoing TB screening

	Total *N* = 1,549	Male *N* = 727	Female *N* = 822	*p*-value
**Demographic and clinical characteristics**				
Age, years	33 (27–40)	36 (30–42)	30 (25–37)	< 0.001
CD4 count, cells/µl	158 (67–263)	139 (62–237)	183 (72–281)	< 0.001
CD4 ≤ 200 cells/µl	606 (39.1%)	242 (33.3%)	364 (44.3%)	< 0.001
BMI, kg/m^2^	21.1 (18.9–23.9)	20.2 (18.4–22.2)	22.3 (19.7–25.2)	< 0.001
Temperature, Celsius	36.4 (36.0-36.7)	36.4 (36.0-36.8)	36.4 (36.1–36.7)	0.78
Heart rate, bpm	90 (78–103)	87 (75–103)	92 (81–104)	< 0.001
Previous TB	55 (3.6%)	30 (4.1%)	25 (3.0%)	0.15
Any prior opportunistic infection	655 (42.3%)	335 (46.2%)	320 (39.1%)	0.01
Pregnant at baseline	23 (1.5%)	—	23 (2.8%)	—
Household TB contact^1^	208 (14.2%)	98 (14.2%)	110 (14.1%)	0.93
Smoked > 100 cigarettes^2^	221 (15.5%)	206 (31.0%)	15 (2.0%)	< 0.001
Ever alcohol use	1,160 (74.9%)	582 (80.1%)	578 (70.3%)	< 0.001

Abbreviations: IQR, interquartile range; bmp, beats per minute; TB, tuberculosis

^1^ Currently or previously living with someone with TB disease; 80 missing (39 male, 41 female)

^2^ 119 missing (63 male and 56 female)

Legend: Cells represent median (interquartile range [IQR]) or number (%).

**Table 2:** Results of TB screening and testing among ART-naive males and females with HIV

	Total *N* = 1,549	Male *N* = 727	Female *N* = 822	*p*-value
**Symptom screen**				
Symptom screen positive^1^	1,358 (87.7%)	657 (90.4%)	701 (85.3%)	0.002
Current cough	780 (50.4%)	402 (55.3%)	378 (46.0%)	< 0.001
Cough duration, days	14 (7–30)	21 (7–30)	14 (7–30)	0.004
Fever	794 (51.3%)	394 (54.2%)	400 (48.7%)	0.03
Fever duration, days	14 (7–30)	15 (7–30)	14 (7–30)	0.001
Night sweats	538 (34.7%)	316 (43.5%)	222 (27.0%)	< 0.001
Night sweats duration, days	21 (7–30)	21 (7–30)	17 (7–30)	0.65
Weight loss	1,128 (72.8%)	562 (77.3%)	566 (68.9%)	< 0.001
Weight loss > 5 kg	633 (56.1%)	323 (57.5%)	310 (54.8%)	0.36
Number of symptoms	2 (1–3)	2 (1–3)	2 (1–3)	< 0.001
**CRP**				
CRP ≥ 5 mg/L	740 (47.8%)	413 (56.8%)	327 (39.8%)	< 0.001
CRP, mg/L	4.03 (2.50-22.71)	6.17 (2.50-41.37)	2.84 (2.50-10.66)	< 0.001
Symptom screen positive^1^ and CRP ≥ 5 mg/L	704 (45.5%)	398 (54.8%)	306 (37.2%)	< 0.001
**Xpert MTB/RIF**				
Xpert positive	145 (9.4%)	89 (12.2%)	56 (6.8%)	< 0.001
Xpert semi-quantitative very low	59 (40.7%)	32 (35.0%)	27 (48.2%)	0.03
Xpert semi-quantitative low	46 (31.7%)	26 (29.2%)	20 (35.7%)	
Xpert semi-quantitative medium	32 (22.1%)	23 (25.8%)	9 (16.1%)	
Xpert semi-quantitative high	8 (5.5%)	8 (9.0%)	0 (0%)	
**Mycobacterial culture**				
Culture-positive TB	236 (15.2%)	149 (20.5%)	87 (10.6%)	< 0.001
Days-to-positive culture	11 (7–16)	11 (7–17)	12 (8–15)	0.89
Initiated TB treatment	208 (88.1%)	133 (89.3%)	75 (86.2%)	0.53
Days-to treatment initiation	3 (1–32)	2 (0–28)	4 (1–43)	0.13

Abbreviations: IQR, interquartile range; TB, tuberculosis; kg, kilograms; CRP, C-reactive protein

^1^ Presence of cough, fever, night sweats, and/or weight loss

Legend: Cells represent median (interquartile range [IQR]) or number (%)

**Table 3:** Odds of culture positive TB among ART-naïve people with HIV

	Odds Ratio (95% CI)	*p*-value	Adjusted Odds Ratio^1^ (95% CI)	*p*-value
Male sex	2.18 (1.64–2.90)	< 0.001	2.05 (1.40–3.01)	< 0.001
Age, years	1.01 (0.99–1.03)	0.06	1.01 (0.99–1.03)	0.99
Pre-ART CD4 count, cells/µl	0.99 (0.99–0.99)	< 0.001	1.00 (0.99-1.00)	0.12
BMI, kg/m^2^	0.82 (0.78–0.85)	< 0.001	0.90 (0.85–0.95)	< 0.001
Temperature, Celsius	2.47 (2.02–3.03)	< 0.001	1.58 (1.25–2.01)	< 0.001
Heart rate, bpm	1.05 (1.04–1.06)	< 0.001	1.04 (1.03–1.05)	< 0.001
Smoked > 100 cigarettes	1.72 (0.20–2.47)	0.003	1.24 (0.80–1.92)	0.33
Ever alcohol use	1.33 (0.95–1.87)	0.10	—	—

Legend: Age, CD4 count, BMI, temperature, and heart rate were treated as continuous

^1^ Sex, age, CD4 count, BMI, temperature, heart rate, and smoking included in multivariable model

### Measures of mycobacterial load

Compared to females, males reported a greater number of symptoms (current cough, fever, night sweats, weight loss) and longer duration of symptoms (Table 2). Among those with culture-confirmed TB, median CRP levels (median 57.42 vs. 35.55 mg/L, *p* = 0.004) and Xpert semi-quantitative grade (*p* = 0.04) were significantly higher among males than females (Supplementary Table 2); however, there was no difference in the proportion of culture-positive TB cases detected by Xpert between males and females (56.4% vs. 58.6%, *p* = 0.74). In addition, days-to-culture positivity did not differ by sex (median 11 vs. 12, *p* = 0.89, Table 2).

**Table 4:** Diagnostic accuracy of symptom screening, point-of-care C-reactive protein, and Xpert MTB/RIF for culture-confirmed TB among males and females with HIV

	Total *N* = 1,549	Male *N* = 727	Female *N* = 822	Difference, Male vs. Female (95% CI)
**Symptom screening**				
Sensitivity (95% CI)	96.2% (92.9-98.2%)	96.0% (91.4-98.5%)	96.6% (90.3-99.3%)	-0.5% (-5.5%, 4.4%)
Specificity (95% CI)	13.9% (12.0-15.8%)	11.1% (8.6-13.9%)	16.1% (13.5-18.9%)	-5.0% (-8.7%, -1.3%)
Positive predictive value (95% CI)	16.7% (14.8-18.8%)	21.8% (18.7-25.1%)	12.0% (9.7-14.6%)	9.8% (5.8%, 13.8%)
Negative predictive value (95% CI)	95.3% (91.2-97.8%)	91.4% (82.3-96.8%)	97.5% (92.9-99.5%)	-6.1% (-13.2%, 1.0%)
**Point-of-care CRP**				
Sensitivity (95% CI)	90.7% (86.2-94.1%)	89.9% (83.9-94.3%)	92.0% (84.1-96.7%)	-2.0% (-9.5%, 5.5%)
Specificity (95% CI)	59.9% (57.2-62.6%)	51.7% (47.6-55.9%)	66.4% (62.9-69.8%)	-14.7% (-20.0%, -9.3%)
Positive predictive value (95% CI)	28.9% (25.7-32.3%)	32.4% (27.9-37.2%)	24.5% (19.9-29.5%)	8.0% (1.5%, 14.5%)
Negative predictive value (95% CI)	97.3% (95.9-98.3%)	95.2% (92.2-97.3%)	98.6% (97.1-99.4%)	-3.4% (-5.9%, -0.8%)
**Xpert MTB/RIF**				
Sensitivity (95% CI)	57.2% (50.6-63.6%)	56.4% (48.0-64.5%)	58.6% (47.6-69.1%)	-2.2% (-15.3%, 10.8%)
Specificity (95% CI)	99.2% (98.6-99.6%)	99.1% (98.0-99.7%)	99.3% (98.4-99.8%)	-0.2% (-1.2%, 0.8%)
Positive predictive value (95% CI)	93.1% (87.7-96.6%)	94.4% (87.4-98.2%)	91.1% (80.4-97.0%)	3.3% (-5.6%, 12.2%)
Negative predictive value (95% CI)	92.8% (91.3-94.1%)	89.8% (87.2–92.0%)	95.3% (93.6-96.7%)	-5.5% (-8.3%, -2.7%)
**Sequential screening: Symptom screening followed by CRP**,** if symptom screen-positive**				
Sensitivity (95% CI)	89.8% (85.2-93.4%)	89.3% (83.1-93.7%)	90.8% (82.7-95.9%)	-1.5% (-9.4%, 6.3%)
Specificity (95% CI)	62.5% (59.8-65.2%)	54.2% (50.0-58.3%)	69.1% (65.6-72.4%)	-15.0% (-20.2%, -9.7%)
Positive predictive value (95% CI)	30.1% (26.7-33.7%)	33.4% (28.8-38.3%)	25.8% (21.0-31.1%)	7.6% (0.9%, 14.3%)
Negative predictive value (95% CI)	97.2% (95.8-98.2%)	95.1% (92.2-97.2%)	98.4% (97.0-99.3%)	-3.3% (-5.9%, -0.8%)

### TB screening test characteristics

*WHO 4-part symptom screen.* A total of 1,358 (87.7%) participants screened positive for TB by the WHO 4-part symptom screen, including 657/727 (90.4%) males and 701/822 (85.3%) females (*p* = 0.002, Table 2). Prevalence of each individual TB symptom (current cough, fever, night sweats, weight loss) was higher and self-reported symptom duration longer among males compared to females. Sensitivity of symptom screening for TB was similarly high for males and females (96.0% vs. 96.6%, difference − 0.5%, 95% CI -5.5 to 4.4%, Table 4), while specificity was lower for males than females (11.1% vs. 16.1%, difference − 5.0%, 95% CI -8.7% to -1.3%).

*CRP screening.* A total of 740 (47.8%) participants screened positive by CRP (CRP ≥ 5 mg/L), including 413/727 (56.8%) males and 327/822 (39.8%) females (*p* < 0.001, Table 2). Males also had higher baseline CRP levels than females (median 6.17 vs. 2.84 mg/L, *p* < 0.001). Sensitivity of CRP for culture-confirmed TB was lower than that of symptom screening, and was similar for males and females (89.9% vs. 92.0%, difference − 2.0%, 95% CI -9.5 to 5.5%, Table 4). Like the WHO symptom screen, specificity of CRP for TB was lower for males than females (51.7% vs. 66.4%, difference − 14.7%, 95% CI -20.0% to -9.3%), however specificity of CRP was substantially higher than symptom screen specificity regardless of sex.

*Xpert screening.* A total of 145 (9.4%) participants screened positive for TB via Xpert, including 89/727 (12.2%) males and 56/822 (6.8%) females (*p* < 0.001, Table 2). Sensitivity of Xpert for TB was substantially lower than both symptom screening and CRP, but was similar for males and females (56.4% vs. 58.6%, difference − 2.2%, 95% CI -15.3 to 10.8%, Table 4). Specificity of Xpert for TB was substantially higher than symptom screening and CRP, and was similar for males and females (99.1% vs. 99.3%, difference − 0.2%, 95% CI -1.2 to 0.8%).

*WHO symptom screen*,* followed by CRP.* A total of 704 (45.5%) participants screened positive for TB (positive by both symptoms and CRP), including 398/727 (54.8%) males and 306/822 (37.2%) females (*p* < 0.001, Table 2). Sensitivity of sequential screening with symptoms followed by CRP was similar for males and females (89.3% vs. 90.8%, difference − 1.5%, 95% CI -9.4 to 6.3%), while specificity was lower for males than females (54.2% vs. 69.1%, difference − 15.0%, 95% CI -20.2% to -9.7%, Table 4). There was no difference in sensitivity (*p* = 0.76) or specificity (*p* = 0.17) between this combination screening strategy and screening based on CRP levels alone.

### TB treatment initiation and early mortality

*TB treatment initiation.* Among 236 participants with culture-confirmed TB, 208 (88.1%) initiated TB treatment, including 133/149 (89.3%) males and 75/87 (86.2%) females (*p* = 0.53, Table 2). Median days-to-treatment initiation was similar for males and females (2 vs. 4, *p* = 0.13). Of the 28 participants with culture-confirmed TB who did not initiate TB treatment, 5 (17.9%) declined to initiate TB treatment (4 females and 1 male). TB treatment status was unknown for 23 participants, including 8 (28.6%) who died within three months of enrollment (4 males and 4 females, median time-to-death 35 days [IQR 22–42]) and 9 (32.1%) who were lost to follow-up (5 males and 4 females).

*Early mortality.* A total of 48 (3.1%) participants died within three months of enrollment, including 29/727 (4.0%) males and 19/822 (2.3%) females (*p* = 0.06). Among the 236 participants with culture-confirmed TB, 22 (9.3%) died within three months, including 14/149 (9.4%) males and 8/87 (9.2%) females (*p* = 0.96). Among 1,313 participants without culture-confirmed TB, 26 (2.0%) died within three months, including 15/578 (2.6%) males and 11/735 (1.5%) females (*p* = 0.16).

In a sensitivity analysis assuming that all 9 participants with culture-confirmed TB who were lost to follow-up died within three months of enrollment, there was no difference in three-month mortality by sex among those with culture-positive TB (31/149 [20.8%] males vs. 26/87 [29.9%] females, *p* = 0.12).

## Discussion

In this study evaluating sex differences in ART-naïve adults with HIV undergoing clinic-based systematic screening for TB in Uganda, we found that males presented with more advanced HIV and had two-fold higher TB prevalence than females. Among those with culture-confiremed TB, males had higher median CRP levels and other measures of mycobacterial load at the time of diagnosis than females (which are predictors of poor TB outcomes [20, 21]). However, despite males presenting with these initial disadvantages, time-to-TB treatment initiation and 3-month mortality were similar for males and females co-infected with TB. These results suggest that while males with HIV have excess TB prevalence and higher mycobacterial load than females, systematic screening may reduce sex differences in early TB mortality.

It is well-established that compared to females, males experience elevated TB prevalence (accounting for up to 75% of TB cases worldwide) and TB-associated morbidity and mortality [1, 5]. In our study, we confirmed that TB prevalence among males with HIV undergoing clinic-based systematic TB screening was approximately two-fold higher than females (20.5% vs. 10.6%) and that males co-infected with TB had higher mycobacterial load (as measured by Xpert) at the time of TB diagnosis. These results are consistent with data from TB prevalence surveys, which demonstrate higher TB prevalence and higher prevalence of smear-positive disease among males compared to females in high HIV burden settings [2, 3]. The higher TB burden observed in males worldwide has long been assumed to stem from social and behavioral factors that can lead to increased risk of TB exposure and infection (e.g., more time spent outside the home, a larger number of social contacts, and more occupational exposure), progression to TB disease (e.g., higher prevalence of smoking and alcohol consumption), and poor TB outcomes (e.g., delayed presentation to care, poorer ART and TB treatment adherence) for men compared to women [22]. Our results align with these trends, demonstrating a greater burden of behavioral TB risk factors and characteristics suggestive of delayed entry into care for males: In our study, males were older, had lower pre-ART CD4 counts, and reported more opportunisitic infections, TB symptoms, and longer symptom duration compared to females. Furthermore, males had higher prevalence of smoking and alcohol use than females. Importantly, despite presenting with more advanced HIV and TB, once diagnosed with TB, males in our study initiated treatment equally promptly and had similar short term outcomes to females. This suggests that clinic-based systematic screening has the potential to attenuate sex-based disparities in early TB mortality by reducing diagnostic and treatment delays once individuals have engaged in care, which may be particularly important for males with TB.

In addition to social and behavioral factors, biologic differences between males and females likely help drive sex differences in TB among PWH, yet are poorly understood. Previous studies have shown higher bacillary burden and mortality in male mice following *M. tuberculosis* infection [23–25] and have demonstrated the influence of sex hormones on mycobacterial growth and immune responses [26–30], which may contribute to more severe disease in males compared to females. However, biologic sex differences in TB remains understudied, and the additional impact of HIV co-infection is unclear. Our finding that clinic-based systematic screening may reduce differences in TB outcomes between males and females with HIV should prompt additional research into the biological factors that contribute to sex differences in TB disease and prognosis for PWH, and their interaction with the social and behavioral factors that also drive these outcomes.

As of 2021, health care providers have more options to screen for TB disease in PWH. In addition to symptom screening and CXR, WHO guidelines now support the use of CRP and Xpert for systematic TB screening among PWH [6]. However, despite differences in sensitivity and specificity of screening tools across different populations of PWH (e.g., ART-naïve vs. on ART) and variability in costs and resource requirements for implementation, current guidelines do not outline when to choose one screening tool over another [6]. Here, we found that symptom screening, CRP (using a 5 mg/L cut-point), and symptoms + CRP met or exceeded the 90% sensitivity target established by the WHO for an effective TB screening test among ART-naïve males and females. In contrast, the standard Xpert cartridge failed to meet this threshold when culture was used as the reference standard for both males (sensitivity 56%) and females (sensitivity 59%). Although Xpert MTB/RIF Ultra (Ultra) has higher sensitivity than the standard cartridge, sensitivity of Ultra in the context of active case finding (~ 70%) still fails to meet the 90% target [31, 32]. Our results support the use of WHO-recommended TB screening strategies for PWH entering care, with similar sensitivies for males and females.

Although our results show that males and females with culture-positive TB are equally likely to screen-positive via symptom screening or CRP (with approximately 96% of males and females with TB identified through symptom screening and 91% through CRP-based screening), previous studies have demonstrated that the quality of routine TB evaluation is worse for women than men in many settings, with women less likely to be referred for confirmatory TB testing following a positive screening test result [33–35]. Thus, implementation of rigorous, systematic TB screening and testing protocols in health care settings remains important to minimize sex disparities in detection of TB among PWH.

Importantly, specificity of symptom screening, CRP, and symptoms + CRP failed to meet the minimum 70% target recommended by WHO, and was markedly lower in males than females, potentially due to a higher prevalence of non-TB disease among males, which may arise from the same gendered social and behavioral factors that contribute to males entering HIV care with more advanced HIV and TB. Maximizing specificity of TB screening (while maintaining high sensitivity) is important for facilitating prompt initiation of TB preventive therapy for PWH without TB disease without the need for unnecessary and costly confirmatory TB testing [36]. In our study, the reduced specificity of symptom screen, CRP, and symptom screen + CRP among males suggests that, relative to females, males may be more likely to miss opportunities to initiate TB preventive therapy using these screening strategies. Differences in specificity were less pronounced for symptom-based screening (difference 5%) than CRP (difference 15%) or a combination strategy (difference 15%), likely because the majority of both males (90%) and females (85%) screened positive by symptoms. While sex differences in CRP levels have been reported previously for PWH entering care [37, 38], our results expand on these findings and suggest that while differences in CRP levels between males and females are unlikely to result in differential TB case detection, they could lead to lower TB preventive treatment uptake among males. Importantly, CRP-based screening had substantially higher specificity compared to symptom screening among both males (52% vs. 11%) and females (66% vs. 16%), reaffirming the utility of CRP alone as a TB screening tool in this population.

This study has several limitations. First, we did not assess several important parameters, including socioeconomic status, occupational history, and biologic factors that may have contributed to our observed outcomes. Future studies that rigorously investigate both epidemiologic and biological sex differences in TB for PWH are needed. Second, we included only people with CD4 counts ≤ 350 cells/uL who were ART-naïve. People with advanced HIV disease not receiving ART are at greatest risk of TB and represent a critical population in need of screening; however, PWH receiving ART remain at elevated TB risk compared to HIV-negative individuals and should be screened for TB regularly [6]. Therefore, additional studies evaluating possible sex differences in TB risk, outcomes, and diagnostic accuracy of TB screening tools for individuals with with higher CD4 cell counts and receiving ART should be performed. Third, we only evaluated the diagnostic accuracy of symptom screening, CRP, and Xpert. Symptom screening remains the primary strategy to screen for TB disease in PWH globally, and CRP is a novel screening tool with strong potential to improve uptake of TB preventive therapy [15, 16, 39]. However, sex differences in other TB screening tools and screening algorithms—including CXR and Ultra [6]—should also be assessed. Finally, we identified TB only through evaluation of sputum specimens, and did not perform additional testing to confirm or rule-out extrapulmonary TB. While pulmonary TB represents the majority of cases worldwide, extrapulmonary disease accounts for nearly 20% of TB disease [1], and possible sex differences in extrapulmonary TB should be evaluated.

In conclusion, we found that ART-naïve male PWH with CD4 count ≤ 350 undergoing clinic-based systematic TB screening had double the TB prevalence as females, reaffirming that systematic TB screening and early TB diagnosis is particularly important for this population. Although mycobacterial load was higher among males co-infected with TB, there was no difference in early TB-associated mortality by sex. These results suggest that clinic-based systematic screening has the potential to reduce sex-based disparaties in TB outcomes among PWH. Despite this, males with HIV may be more likely to miss opportunities for TB preventive therapy than females. While identifying novel screening tools with sufficiently high sensitivity and specificity will be essential to further reduce the burden of TB among all PWH, expanded systematic TB screening with currently available tools should be considered in high burden settings to more directly address the broader TB epidemic.

## Electronic Supplementary Material

Below is the link to the electronic supplementary material.

Supplementary Material 1

## Data Availability

The datasets used and/or analysed during the current study are available from the corresponding author on reasonable request.

## Abbreviations

ART: Antiretroviral therapy
BMI: Body mass index
CRP: C-reactive protein
CXR: Chest X-ray
HIV: Human immunodeficiency virus
POC: Point of care
PWH: People with HIV
TB: Tuberculosis
WHO: World Health Organization
