# Supplementary material for: Sex differences in systematic screening for tuberculosis among antiretroviral therapy naïve people with HIV in Kampala, Uganda
Source: BMC Infect Dis. 2025 Apr 1;25:452. doi: 10.1186/s12879-025-10835-4 (PMC11963461; doi:10.1186/s12879-025-10835-4)
Supplement: Supplementary file 1 — Supplementary Material 1 [file 12879_2025_10835_MOESM1_ESM.docx]

**Supplementary Table 1.** Characteristics of ART-naive pregnant females with HIV undergoing TB screening

|  | **Total**  **N=23** |
| --- | --- |
| **Demographic and clinical characteristics** |  |
| Age, years | 23 (20-30) |
| CD4 count, cells/μl | 267 (156-322) |
| CD4 ≤200 cells/μl | 16 (69.6%) |
| BMI, kg/m^2^ | 23.9 (21.3-28.9) |
| Previous TB | 0 (0%) |
| Any prior opportunistic infection | 10 (43.5%) |
| Household TB contact^1^ | 4 (17.4%) |
| Smoked >100 cigarettes^2^ | 0 (0%) |
| Ever alcohol use | 16 (69.6%) |
| **Symptom screen** |  |
| Symptom screen positive^3^ | 16 (69.6%) |
| Current cough | 4 (17.4%) |
| Cough duration, days | 11 (5-52) |
| Fever | 6 (26.1%) |
| Fever duration, days | 16 (7-30) |
| Night sweats | 3 (13.0%) |
| Night sweats duration, days | 7 (4-30) |
| Weight loss | 10 (43.5%) |
| Weight loss >5kg | 3 (30.0%) |
| **CRP** |  |
| CRP ≥5 mg/L | 10 (43.5%) |
| CRP, mg/L | 3.03 (2.50-12.94) |
| Symptom screen positive^3^ and CRP ≥5 mg/L | 7 (30.4%) |
| **Xpert** |  |
| Xpert positive | 0 (0%) |
| **Mycobacterial culture** |  |
| Culture positive TB | 0 (0%) |
| *Abbreviations:* IQR, interquartile range; TB, tuberculosis; kg, kilograms; CRP, C-reactive protein  ^1^ Currently or previously living with someone with TB disease  ^2^ 3 missing  ^3^ Presence of cough, fever, night sweats, and/or weight loss  *Legend:* Cells represent median (interquartile range [IQR]) or number (%). | |

**Supplementary Table 2.** Results of TB screening and testing among ART-naive males and females with HIV and culture-confirmed TB

|  | **Total**  **N=236** | **Male**  **N=149** | **Female**  **N=87** | **p-value** |
| --- | --- | --- | --- | --- |
| **Symptom screen** |  |  |  |  |
| Symptom screen positive^1^ | 227 (96.2%) | 143 (96.0%) | 84 (96.6%) | 1.00 |
| Current cough | 174 (73.7%) | 109 (73.2%) | 65 (74.7%) | 0.79 |
| Cough duration, days | 21 (14-30) | 21 (14-30) | 21 (14-30) | 0.77 |
| Fever | 178 (75.4%) | 116 (77.9%) | 62 (71.3%) | 0.26 |
| Fever duration, days | 21 (7-30) | 21 (14-30) | 14 (7-30) | 0.04 |
| Night sweats | 139 (58.9%) | 92 (61.7%) | 47 (54.0%) | 0.25 |
| Night sweats duration, days | 21 (7-30) | 26 (7-30) | 14 (7-30) | 0.12 |
| Weight loss | 212 (89.8%) | 135 (90.6%) | 77 (88.5%) | 0.61 |
| Weight loss >5kg | 162 (76.4%) | 104 (77.0%) | 58 (75.3%) | 0.78 |
| **CRP** |  |  |  |  |
| CRP ≥5 mg/L | 214 (90.7%) | 134 (89.9%) | 80 (92.0%) | 0.65 |
| CRP, mg/L | 49.52 (18.25-95.86) | 57.42 (25.17-101.35) | 35.55 (12.27-68.60) | 0.004 |
| Symptom screen positive^1^ and  CRP ≥5 mg/L | 212 (89.8%) | 133 (89.3%) | 79 (90.8%) | 0.83 |
| **Xpert MTB/RIF** |  |  |  |  |
| Xpert positive | 135 (57.2%) | 84 (56.4%) | 51 (58.6%) | 0.74 |
| Xpert semi-quantitative very low | 50 (37.0%) | 28 (33.3%) | 22 (43.1%) | 0.04 |
| Xpert semi-quantitative low | 45 (33.3%) | 25 (29.7%) | 20 (39.2%) |  |
| Xpert semi-quantitative medium | 32 (23.7%) | 23 (27.4%) | 9 (17.7%) |  |
| Xpert semi-quantitative high | 8 (5.9%) | 8 (9.5%) | 0 (0%) |  |
| *Abbreviations:* IQR, interquartile range; TB, tuberculosis; kg, kilograms; CRP, C-reactive protein  ^1^ Presence of cough, fever, night sweats, and/or weight loss  *Legend:* Cells represent median (interquartile range [IQR]) or number (%) | | | | |

**Supplementary Table 3.** Results of TB screening among ART-naive males and females with HIV who were culture-negative

|  | **Total**  **N=1,313** | **Male**  **N=578** | **Female**  **N=735** | **p-value** |
| --- | --- | --- | --- | --- |
| **Symptom screen** |  |  |  |  |
| Symptom screen positive^1^ | 1,131 (86.1%) | 514 (88.9%) | 617 (84.0%) | 0.01 |
| Current cough | 606 (46.2%) | 293 (50.7%) | 313 (42.6%) | 0.003 |
| Cough duration, days | 14 (7-30) | 14 (7-30) | 14 (7-30) | 0.003 |
| Fever | 616 (46.9%) | 278 (48.1%) | 338 (46.0%) | 0.45 |
| Fever duration, days | 14 (7-30) | 14 (7-30) | 14 (7-30) | 0.06 |
| Night sweats | 399 (30.4%) | 224 (38.8%) | 175 (23.8%) | <0.001 |
| Night sweats duration, days | 21 (7-30) | 21 (7-30) | 21 (7-30) | 0.84 |
| Weight loss | 916 (69.8%) | 427 (73.9%) | 489 (66.5%) | 0.004 |
| Weight loss >5kg | 471 (51.4%) | 219 (51.3%) | 252 (51.5%) | 0.94 |
| **CRP** |  |  |  |  |
| CRP ≥5 mg/L | 526 (40.1%) | 279 (48.3%) | 247 (33.6%) | <0.001 |
| CRP, mg/L | 2.83 (2.50-10.18) | 4.15 (2.50-18.33) | 2.50 (2.50-6.79) | <0.001 |
| Symptom screen positive^1^ and  CRP ≥5 mg/L | 492 (37.5%) | 265 (45.9%) | 227 (30.9%) | <0.001 |
| *Abbreviations:* IQR, interquartile range; TB, tuberculosis; kg, kilograms; CRP, C-reactive protein  ^1^ Presence of cough, fever, night sweats, and/or weight loss  *Legend:* Cells represent median (interquartile range [IQR]) or number (%) | | | | |
